# Developing the NewAthena X-IFU Cryogenic AntiCoincidence Detector (CryoAC): From Microfabrication Process Standardization to Cryogenic Functional Verification Toward TRL5

**DOI:** 10.3390/s26154985

**Published:** 2026-08-06

**Authors:** Claudio Macculi, Matteo D’Andrea, Giacomo Gorla, Simone Lotti, Gabriele Minervini, Francesco Monastra, Luigi Piro, Lorenzo Ferrari Barusso, Edvige Celasco, Flavio Gatti, Daniele Grosso, Manuela Rigano, Fabio Chiarello, Guido Torrioli, Mauro Fiorini, Michela Uslenghi, Daniele Brienza, Elisabetta Cavazzuti, Chiara Grappasonni, Simonetta Puccetti, Angela Volpe, Paolo Bastia, Artur Cardoso Coimbra, Francesco Villa

**Affiliations:** 1 INAF/IAPS Roma, Via del Fosso del Cavaliere 100, 00133 Rome, Italy; matteo.dandrea@inaf.it (M.D.); giacomo.gorla@inaf.it (G.G.); simone.lotti@inaf.it (S.L.); luigi.piro@inaf.it (L.P.); 2 Physics Department, Sapienza University of Rome, Piazzale Aldo Moro 2, 00185 Rome, Italy; francesco.monastra@inaf.it; 3 INAF HQ, Viale del Parco Mellini 84, 00136 Roma, Italy; gabriele.minervini@inaf.it; 4 INAF/OAR, Via Frascati 33, Monte Porzio Catone, 00040 Rome, Italy; 5 INAF/OAB, Via Brera 28, 20121 Milano, Italy; lorenzo.ferrari@ge.infn.it; 6 Physics Department and INFN Genova, Università di Genova, Via Dodecaneso 33, 16146 Genova, Italy; edvige.celasco@unige.it (E.C.); flavio.gatti@ge.infn.it (F.G.); grosso@fisica.unige.it (D.G.); manuela.rigano@ge.infn.it (M.R.); 7 CNR/IFN Roma, Via del Fosso del Cavaliere 100, 00133 Rome, Italy; fabio.chiarello@cnr.it (F.C.); guido.torrioli@cnr.it (G.T.); 8 INAF/IASF Milano, Via Alfonso Corti 12, 20133 Milano, Italy; mauro.fiorini@inaf.it (M.F.); michela.uslenghi@inaf.it (M.U.); 9 ASI, Via del Politecnico snc, 00133 Rome, Italy; daniele.brienza@asi.it (D.B.); elisabetta.cavazzuti@asi.it (E.C.); chiara.grappasonni@asi.it (C.G.); simonetta.puccetti@asi.it (S.P.); angela.volpe@asi.it (A.V.); 10 Thales Alenia Space Italia S.p.A., Via Enrico Mattei, 1, Gorgonzola, 20064 Milano, Italy; paolo.bastia@thalesaleniaspace.com (P.B.); artur.cardosocoimbra@thalesaleniaspace.com (A.C.C.); francesco.villa@thalesaleniaspace.com (F.V.)

**Keywords:** Athena mission, X-IFU, CryoAC, Transition Edge Sensors, microfabrication, pulsed laser deposition, SQUID, cryogenic testing

## Abstract

**Highlights:**

**What are the main findings?**

- Stable manufacturing protocols with a DM production yield of 66%
- Functional DM performance compliant with the requirements, including a low-energy threshold of ~0.6 keV, and a low power consumption of 5.15 nW per pixel

**What are the implications of the main findings?**

- Successful completion of all the TRL5 demonstration activities

**Abstract:**

The Cryogenic Anticoincidence (CryoAC) detector is a critical subsystem designed to reduce the particle background for the X-ray Integral Field Unit (X-IFU) instrument onboard the NewAthena space observatory, the next ESA X-ray Large mission. Advancing this technology to Technology Readiness Level 5 (TRL5) requires a unified validation spanning both cleanroom microfabrication repeatability and mK low temperature operational performance. This work presents the complete development cycle of the Demonstration Model 1.2 (DM 1.2), which is aimed at completing the TRL5 demonstration path featured by all the critical technologies operating simultaneously. First, single-process verification protocols were established for Iridium pulsed laser deposition, Reactive Ion Etching (RIE), and deep silicon trenching via the Bosch process. Second, three identical single-pixel prototypes were fabricated and subjected to mK characterization. Four-wire resistance measurement results confirmed a 2/3 production yield against strict design targets (T_C_ ~100 mK). Finally, functional testing at a bath temperature of 50 mK using a VTT FAB4 SQUID readout demonstrated excellent performance, including a low-energy threshold of ~0.6 keV, a pixel power dissipation of 5.15 nW, and an energy resolution ΔE_FWHM_ = 735 eV at 6 keV. These combined achievements successfully validate the entire manufacturing and operational baseline against all primary space mission requirements. This paper has to be considered as a review of the CryoAC technology path toward the TRL5 achievement; main findings will be reported and discussed. Details are relegated to other papers.

## 1. Introduction

The ESA next generation X-ray space observatory, NewAthena [1], currently in Phase B for a launch planned in 2039, will operate in a Lagrangian L1 orbit. It will address key questions in Astrophysics, such as the following:

- How and why does ordinary matter assemble into the structures (galaxies, galaxy groups, and galaxy clusters) that we see today?
- How do black holes grow and shape their environment, as well as the cosmological evolution of the galaxies hosting them?

**Figure 1:**
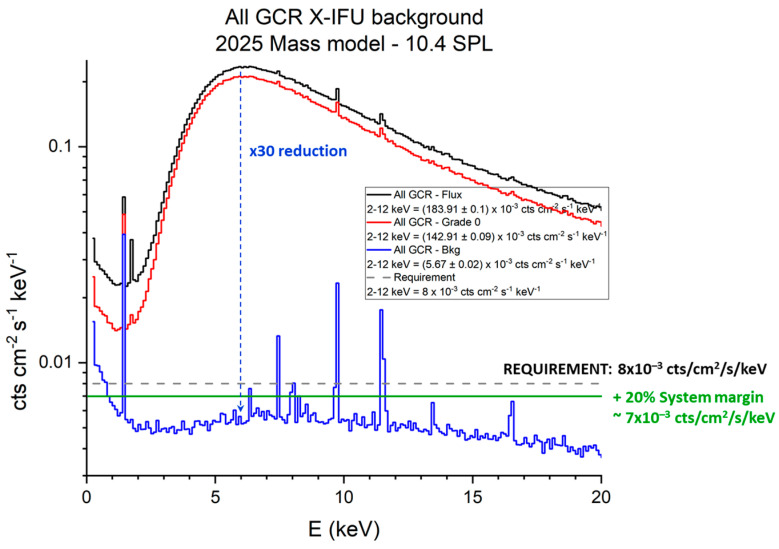
Residual X-IFU NXB as assessed by Geant4 simulation. The black line is the raw particle flux on the main array; the red line are single pixel events representing the main array auto-rejection capability; the blue line is the residual level after the anticoincidence operation. Finally, horizontal lines represent the average level increased by the 20% system margin, and the mission requirement. The results here reported is an update of the ones discussed in Ref. [5] due to a recent revision of the X-IFU mass model. More details in Ref. [7].

It is constituted by two complementary instruments placed at the focus of large area X-ray optics [2], as follows: the WFI [3], and the X-IFU [4], the latter being the reference one for this paper. In this space environment, instruments are subjected to a flux background of Galactic Cosmic Rays (GCR) [5], which are composed primarily of protons, electrons, and alpha particles. The X-IFU FPA (Focal Plane Assembly) houses the main Transition Edge Sensor (TES) microcalorimeter array which is a high-grade imaging spectrometer targeting an energy resolution of 4eV@7keV [6]. This main array cannot distinguish between target astronomical X-ray photons and background cosmic particles, the latter generating the so-called NXB (Non X-ray Background), as follows: the higher the NXB the lower the instrument sensitivity. In the absence of an active background reduction subsystem, Geant4 modeling of the residual NXB shows that a large fraction of the X-IFU observing time would be thoroughly dominated by particle background. To maintain the flagship science return of the observatory, the background flux over the 2–12 keV range must be suppressed. The instrument design sets a strict operational background requirement of 8 × 10^−3^ cts/cm^2^/s/keV. Incorporating a 20% system margin to the result from Geant4 simulation, the expected NXB level is ~7 × 10^−3^ cts/cm^2^/s/keV (see Figure 1). To achieve this constraint is necessary to reduce the background by a factor of ~30 across the main array, a task mainly performed by the Cryogenic AntiCoincidence (CryoAC) detector which is the focus of the present paper.

**Figure 2:**
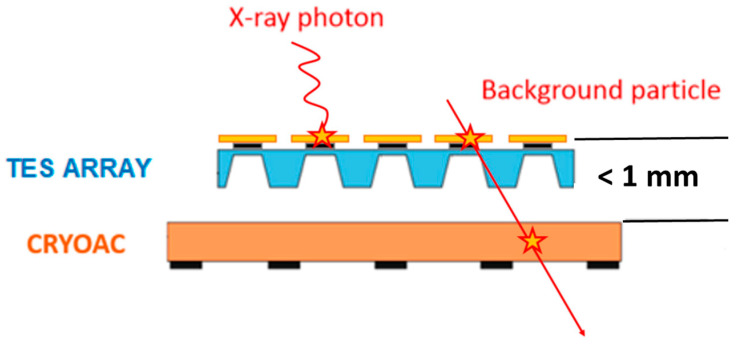
Sketch of the CryoAC vs. TES array detetction plane: the distance has to be lower than 1 mm. While a target X-ray photon is absorbed inside the TES array, a particle crosses both the detection planes thus generating a trigger in the CryoAC detector.

The CryoAC Cold Stage assembly is integrated directly inside the X-IFU FPA at 50 mK. To achieve tight geometric coupling and eliminate geometric escape paths for incoming particles, conditions aimed at increasing the geometrical rejection efficiency of primary particles, the CryoAC is placed at a distance of <1 mm directly below the main TES array sensor (see Figure 2).

**Figure 3:**
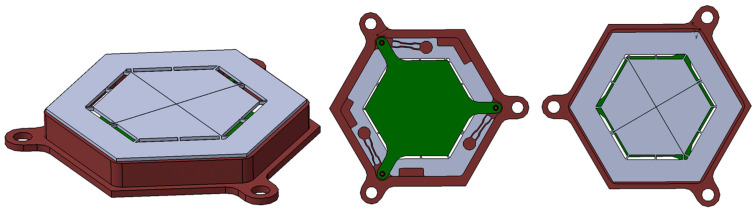
CryoAC Cold Stage assembly concept: in brown the bracket (at center figure the leaf-springs are clearly visible), in grey the chip, in green the CFEE pcb.

The baseline CryoAC chip consists of 4 separate pixels arranged in a segmented layout (see Figure 3). The device utilizes 4 suspended Silicon (Si) absorbers 500 μm thick. The chip has a total active area of ~3.5 cm^2^, each individual pixel having a surface area < 1 cm^2^. Thermal sensing is provided by four individual Ir/Au TES films. Each pixel absorber has an on-board deposited heater for pixel calibration and diagnostics. The chip is mechanically secured inside the FPA using a gold-plated Oxygen-Free High-Conductivity Copper (OFHC Cu) support frame (i.e., bracket), where it is fixed at exactly three independent points by glue onto specialized mechanical “leaf-springs” to mitigate both launch-induced structural loads and thermal contraction at cold. The interface Cold Front End Electronics (CFEE) hosting SQUID for TES readout, and a thermometer, is fastened via screws onto the bracket (opposite side with respect to the chip).

The operation of the 4-pixel cryogenic detector array relies on four completely independent readout chains. Each chain is coupled to a high-sensitivity series array DC SQUID provided by VTT [8]. The warm electronics is divided into two hardware units having different functions, as follows:

- Warm Front-End Electronics (WFEE). This analog subsystem directly drives the cold section. It provides the highly stable DC bias currents needed by both the TES sensors and the SQUIDs. It implements the Flux Locked Loop (FLL) control loops to linearize the SQUID outputs and conditions the raw scientific output signals before digitization
- Warm Back-End Electronics (WBEE). This digital processing block manages high-level data handling. It features on-board DC-DC power isolation converters and performs high-speed digitization of the incoming WFEE scientific analog pulses. The digital logic applies real-time scientific time stamps to the detected events, executes automated trigger identification algorithms and expertise modes, generates telemetry packets, parses Telemetry/Telecommand (TM/TC) instructions, and coordinates WBEE cold redundancy modes

The technical maturity of this electronics design has been verified using fully functional warm electronic breadboards (WFEE BB and WBEE BB) developed under industrial contracts with Thales Alenia Space (TAS-I Milano). These boards use flight-equivalent component grades and are actively integrated into the INAF-IAPS test facilities.

Further, concept cold stage assembly design and validation of the end-to-end simulator at a level of TRL4 have been verified at Demonstration Models (DMs) level, and CryoAC DM samples (1.0, 1.1) have been successfully operated in combination with TES array DM prototypes [9,10,11,12]. The manuscript related to the results of CryoAC/main detector DM1.1 joint operation in DM1.1 FPA is in preparation.

To guarantee strict repeatability across successive CryoAC chip production runs and establish an objective path toward TRL5 [13], the manufacturing procedures [14,15] were systematically updated in coordination with the Italian Space Agency Product Assurance (ASI PA) through a technology readiness assessment activity. The outcome has been the issue of single-process standard manufacturing procedures. Then, a batch of three identical Demonstration Model 1.2 (DM 1.2) samples (#1, #2, and #3) was produced to assess process repeatability and production yield. Each DM1.2 model is a single pixel detector, with a squared absorber, and is aimed at closing all the demonstration activity to get the TRL5.

This paper covers the entire chip development loop aiming at the TRL5, as follows: from the development of microfabrication criteria in the cleanroom at University of Genova, phys. Dpt., to the exhaustive cryogenic campaign carried out at INAF/IAPS Roma.

## 2. DM1.2 Materials and Methods

This section reports some information to frame the TRL5 activity spanning from chip architecture and manufacturing workflow to environmental controls during fabrication and test methodology.

### 2.1. Chip Architecture and Manufacturing Workflow

**Figure 4:**
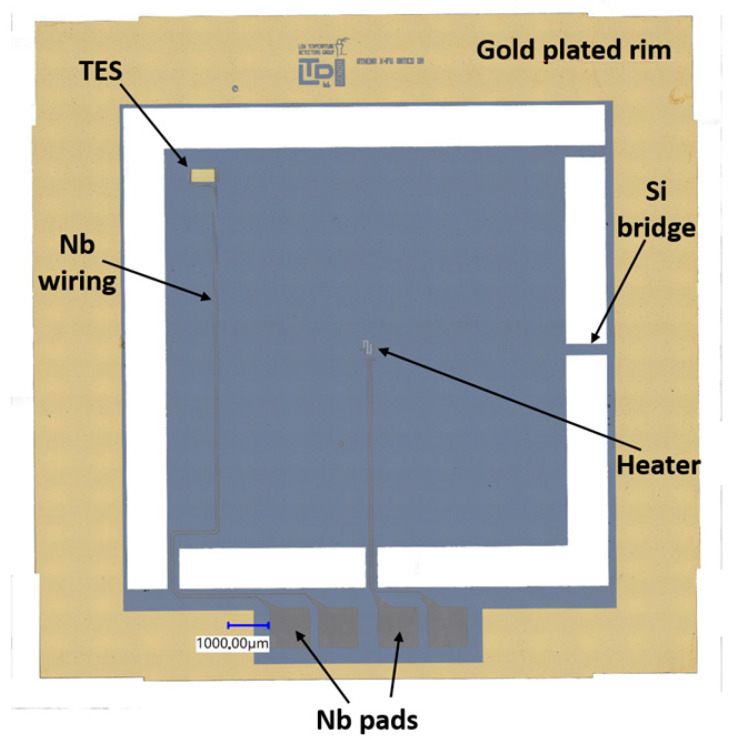
DM1.2 sample #3.

The DM 1.2 CryoAC prototype is a single-pixel detector based on a 500 μm thick silicon squared absorber with an active area of 1 cm^2^ (Figure 4). The absorber is suspended from a gold-plated rim via four narrow silicon bridges (300 μm wide, 1 mm length) to establish a highly reproducible thermal conductance toward the thermal bath. Thermal sensing is provided by a single Iridium/Gold (Ir/Au) Transition Edge Sensor (TES) having area of 320 × 640 μm^2^ and thickness 150/50 nm (Ir/Au) positioned in the bridge-free corner of the absorber. A single platinum (Pt) heater is deposited at the center of the absorber for calibration and diagnostic tasks. Its design is based on the DM1.1 prototype better discussed in reference [14].

**Figure 5:**
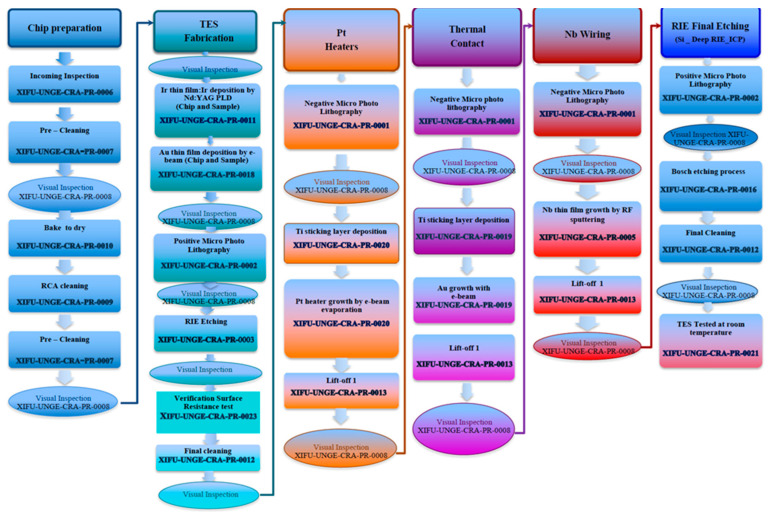
CryoAC DM Chip Manufacturing Process Flow. Each reported code identifies in unambiguous way the related procedure to be carried out.

**Figure 6:**
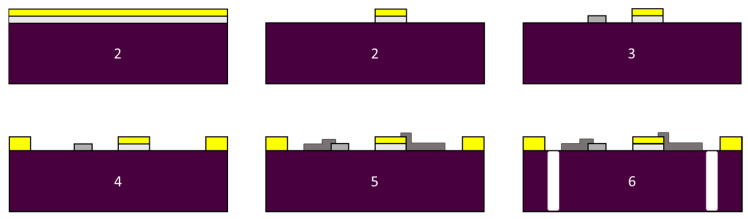
Schematic view of the chip in cross-section. About the numbering, see the bullets in previous page. The big rectangle at the base is the Si wafer, white and yellow thin one is the Ir and Au layers, respectively, light gray is the heater, dark grey the nb wiring.

The complete production chain consists of six highly interdependent microfabrication stages as shown in Figure 5 and Figure 6:

(1) Chip Preparation: Incoming inspection, pre-cleaning, baking, and strict RCA cleaning
(2) TES Fabrication: Nd:YAG Pulsed Laser Deposition (PLD) of Iridium (Ir) thin films and e-beam deposition of Gold (Au), TES RIE
(3) Pt Heaters: Negative photolithography, Titanium (Ti) sticking layer deposition, Platinum (Pt) e-beam evaporation, and lift-off
(4) Thermal Contact: Negative photolithography, Ti sticking layer deposition, and Au e-beam growth
(5) Nb Wiring: RF sputtering of Niobium (Nb) for superconducting electrical wiring
(6) RIE Final Etching: Deep Reactive Ion Etching (DRIE) via the Bosch process for structural silicon definition

In particular, the following critical processes have been verified as from our “X-IFU CryoAC technology demonstration plan” (internal report, unpublished), because deemed critical in enabling the full CryoAC technology, as follows:

- Ir film deposition by PLD, process impacting on active area increase
- Ir/Au TES patterning by positive photolithography and dry etching, process impacting on R-T transition reproducibility
- Si etching by DRIE Bosch process, process impacting on wide area suspended absorbers

After each main process, a visual inspection is performed not only to verify the result of such a process, but also to verify the cleanliness to avoid contaminants, as particles for example, that could negatively affect the following process. To guarantee total traceability, all active tasks were executed under specific standard operating procedures, updated for the TRL5 baseline.

### 2.2. Environmental Controls and Test Methodology

**Figure 7:**
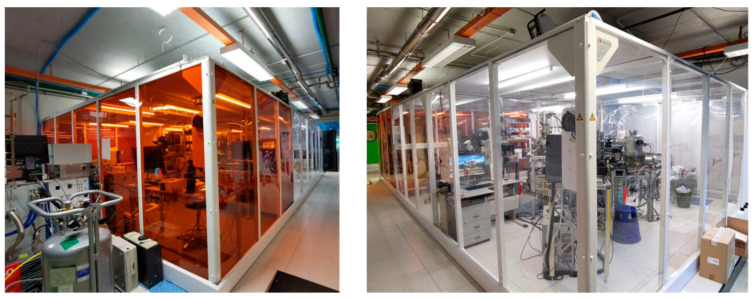
Cleanroom at Genova University, Physics dpt, devoted to NewAthena CryoAC chip production.

Process stability was enhanced by implementing continuous environmental logging (temperature and humidity) during active microfabrication, coupled with real-time monitoring of cleanroom particulate concentrations, the latter performed at an early phase in collaboration with CNES during the cleanroom verification test (see Figure 7).

**Figure 8:**
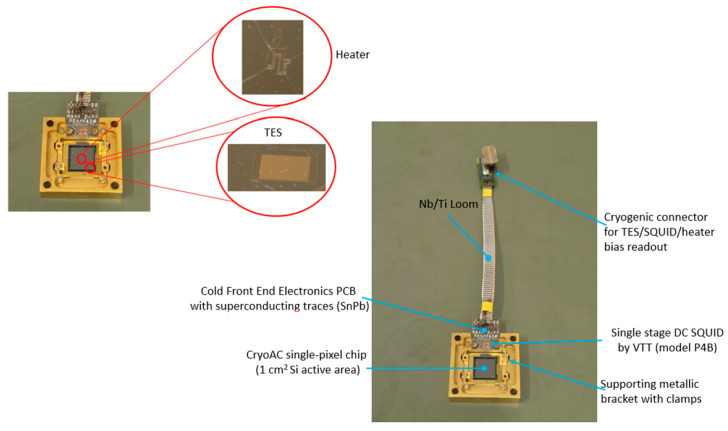
The DM1.2 sample #3 integrated in the holder structure developed for the test in the INAF/IAPS cryogenic setup.

The characterisation of the detector following its manufacturing process was divided into two stages, as follows:

- Four-wire resistance screening performed using a Lakeshore 3570 AC Resistance Bridge (from Lake Shore Cryotronics, Inc., Westerville, OH, USA) to extract TES transition parameters (critical temperature T_C_, transition width ΔT, and normal resistance R_N_), and heater resistance at 50 mK operating temperature (R_H_)
- SQUID Functional Readout: Sample #3 was integrated into an ad hoc cryogenic holder (see Figure 8). The frontend electronics featured a Cold Front End Electronics (CFEE) PCB mated to a single-stage VTT FAB4 DC SQUID (model Q4B rH, from VTT Technical Research Centre of Finland, Espoo, Finland) with an on-board shunt resistor (R_2_ = 7.859 ± 0.005 mΩ at 50 mK) used in the TES bias circuit. The SQUID was operated in a Flux Locked Loop (FLL) configuration using commercial Magnicon XXF-1 electronics (from Magnicon GmbH Barkhausenweg, Hamburg, Germany).

## 3. Results

This section is divided into 3 subsections aimed at showing results from single process verification, production yield, 4-wire transition measurement results, and sample #3 performance results.

### 3.1. Cleanroom Single-Process Verification

As reported in the previous section, before assembling full devices, individual critical steps in production process were isolated and qualified.

#### 3.1.1. Iridium Deposition

**Figure 9:**
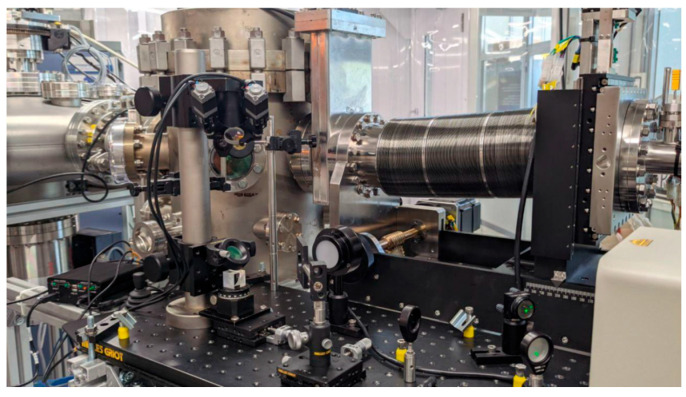
Experimental setup (profilometer Sensofar S neox) to measure the thickness of the deposited Ir thin film over the target 1.5 cm^2^ layout area.

Five separate films were grown via Nd:YAG PLD targeting an average thickness of 143 nm, measured by means of a profilometer (Figure 9) on a reference area of 1.5 cm^2^. The process achieved a high thickness uniformity, i.e., the maximum and minimum thicknesses of the Area of Uniformity (AoU) deviate from the average value by up to 5%, and an AoU > 1.5 cm^2^ for all validated runs, yielding a 4/5 sample success rate as required from the Technology Demonstration Plan.

The following Table 1 reports the results.

#### 3.1.2. TES Etching and Critical Temperature (T_C_)

**Figure 10:**
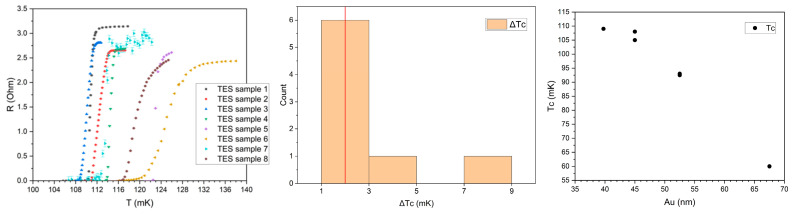
Superconducting transition profiles for the 8 fabricated TES test samples measured at 1 μA, and the corresponding transition width distribution. At right, calibration run introduced to shift T_C_ down to 100 mK by varying the Au content (nominal Ir thickness is 150 nm). The uncertainty on the T_C_ is 1 mK due to the thermometer calibration.

Reactive Ion Etching (RIE) was verified using TES sensor DM-like geometry across a batch of 8 samples structured from 2 primary IrAu bilayers, for a total of 8 TES to be tested at cold. Success criteria have been defined to pass the test. By taking into account the production batch, a yield of at least 6/8 is requested to be considered to have successfully passed the verification campaign. T_C_ are well clustered around a mean value of 116 mK with an overall spread < 20 mK (Figure 10—left; success criterion is <T_C_> ± 10 mK, average over the produced samples; yield obtained 8/8). Transition width maintained tight constraint at ΔT_C_ < 10 mK (Figure 10—center; success criterion is ΔT < 10 mK, yield obtained 8/8). About the normal resistance (R_N_), we evaluated it at an average of 2.7 Ω over the full batch, with individual samples ranging between 2.4 Ω, and 3.1Ω (success criterion of R_N_ within 10% of the average; yield obtained 7/8). All in all, the settled yield is at 7/8 compliant with what is required from the Technology Demonstration Plan. A secondary calibration run was introduced to shift T_C_ down to 100 mK, matching the baseline operational target for the final DM 1.2 sample (Figure 10—right).

#### 3.1.3. Silicon Deep RIE (Si DRIE)

The segmenting step was analyzed across 3 hexagonal silicon test structures using an Inductively Coupled Plasma (ICP) Bosch process. The process achieved a yield of 3 out of 3 samples, meeting the geometric parameters as required from the Technology Demonstration Plan. Note that the trench dividing in 4 pixels the chip is just 25 μm wide (see the cross in Figure 3). The requirements and results of this process are reported in Table 2.

**Figure 11:**
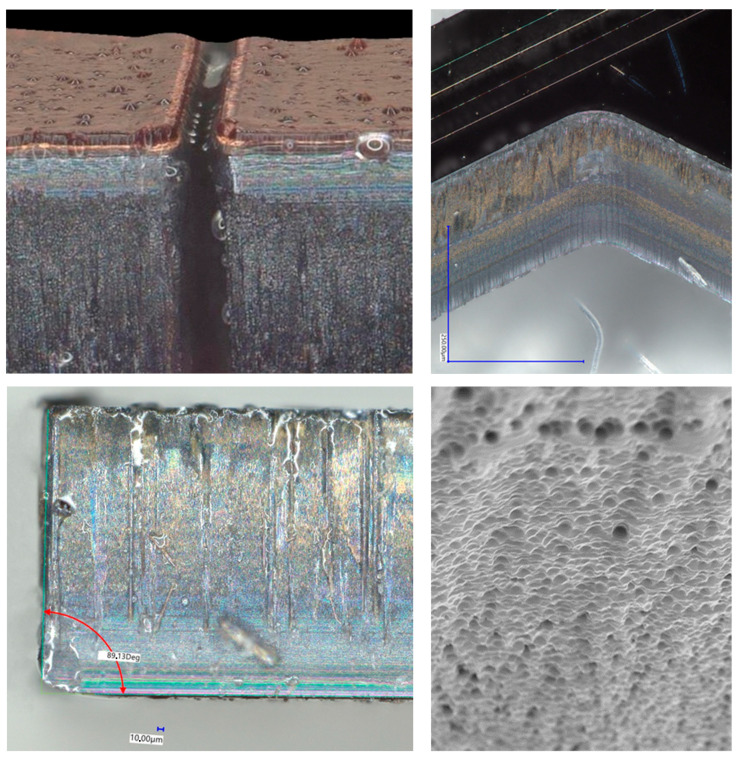
Images showing the microscopic visual inspection of the silicon etching process using the Bosch method. On (**top-left**): the etching mask consists of a photosensitive resin visible on the surface; the process then involves etching the silicon using plasma. The width of the groove must remain constant throughout its entire depth. This is especially true at the bottom, where there must be no widening or narrowing (footing/notching). On (**top-right**): view of one of the bridges from a 40° angle. On (**bottom-left**): view of the wall at one corner to demonstrate the verticality of the etching process. Vertical scars are caused by grass effects and must remain within a 1 μm thickness range. On (**bottom-right**): SEM image of the same wall showing the scalloped structure.

Figure 11 shows examples of the results of a visual inspection under a microscope of the process to demonstrate the etching characteristics.

The completed single-process verification demonstrates that the critical steps in the CryoAC production sequence meet the required performance thresholds. The close grouping of T_C_ profiles around 114 mK verifies the stability of the RIE chemical patterning. Concurrently, the isotropic precision of the Bosch process satisfies the deep trenches required for geometric decoupling of the pixels without inducing unwanted sidewall grass structures.

Following the successful conclusion of the Manufacturing Readiness Review (MRR) with CNES, the manufacturing process procedures are now officially frozen, as follows: everything is now ready to produce the DM1.2 chip as the last step toward the TRL5.

### 3.2. DM1.2 Production Yield and 4-Wire Transition Results

The final TRL5 demonstration has been carried out by using the TRL5 Demonstration Model DM1.2. Three models have been produced and tested to assess yield, as follows: while the performance has been verified on 1 sample, the other 2 samples have only been tested by transition measurement for yield production process (reproducibility).

**Figure 12:**
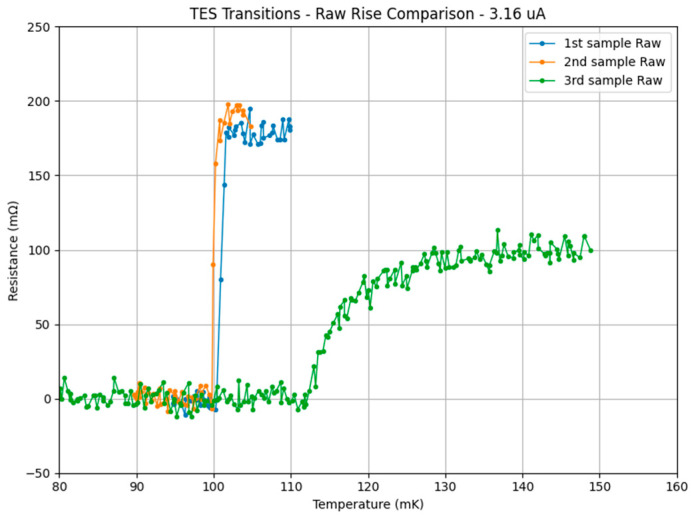
DM1.2 TES transition curve of the three samples obtained by 4-wire measurements.

All three complete DM 1.2 prototypes were cooled down to measure TES properties under a 3.16 μA excitation current. To meet the criteria for standard production yield, samples had to satisfy the design values, as follows: T_C_ = 100 mK ± 10 mK, ∆T < 10 mK, and R_N_ = 200 mΩ ± 10%. As reference, the heater resistance value is R_H_ = 50 Ω (goal), though not to be verified as not being a critical technology as stated in the Technology Demonstration Plan. The resistance of the Pt heater has been measured at 50 mK with an excitation current of 100 nA. The transition curves are shown in Figure 12.

The parameters extracted from the experimental transition curves, and the heater resistance are shown in Table 3 along with the reference values.

Two out of three samples are compliant with the requirements, and thus the production yield is 2/3.

The shift of the critical temperature (Tc) toward higher values observed for sample DM1.2 #3 is most likely due to a higher iridium content in the bilayer, which consequently reduces the proximity effect induced by the gold layer, and the normal resistance as a result of the increased bilayer thickness. The effect is not related to the deposition procedure itself, which has been thoroughly validated (see Section 3.1.1), but rather to the consumption of the Ir pill affecting its surface profile which is not optimal under PLD. As mitigation action, the replacement interval of the pill has been shortened to a maximum of six deposition processes, a threshold within which its surface profile remains optimal.

### 3.3. Functional and Performance Test of CryoAC DM1.2#3 in SQUID Setup

This section is divided into two subsections. First, the best detector working point to carry out a performance test is experimentally determined, then the main results extracted from in-depth testing activity are shown.

In spite of its name, which is merely an internal numbering, sample #3 was the first one delivered to the laboratory. Given the time span of the testing activity and the schedule reporting the milestone to be accomplished in time for TRA, propedeutic for the Mission Adoption Review, we decided to operate such a detector despite the R-T measurement result.

#### 3.3.1. Working Point Definition

**Figure 13:**
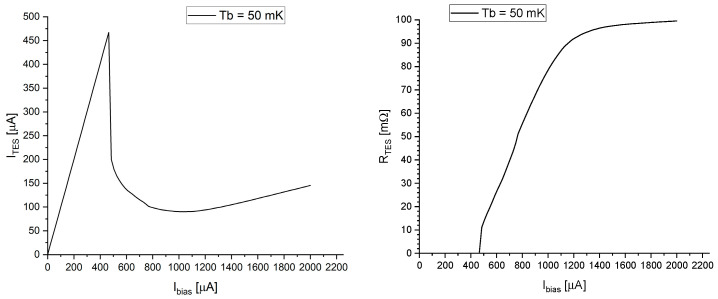
(**Left**) Characteristic I–V curves of the DM1.2 sample #3. (**Right**) RTES vs. IBIAS evaluated from the I–V curve.

Sample #3 was selected for in-depth functional and performance testing using a SQUID in FLL configuration. Current-voltage sweeps (I_TES_ vs. I_bias_) recorded at 50 mK (reference bath temperature for X-IFU) provided well-shaped profiles, establishing that the pixel can be safely biased down to such a base temperature (see Figure 13).

**Figure 14:**
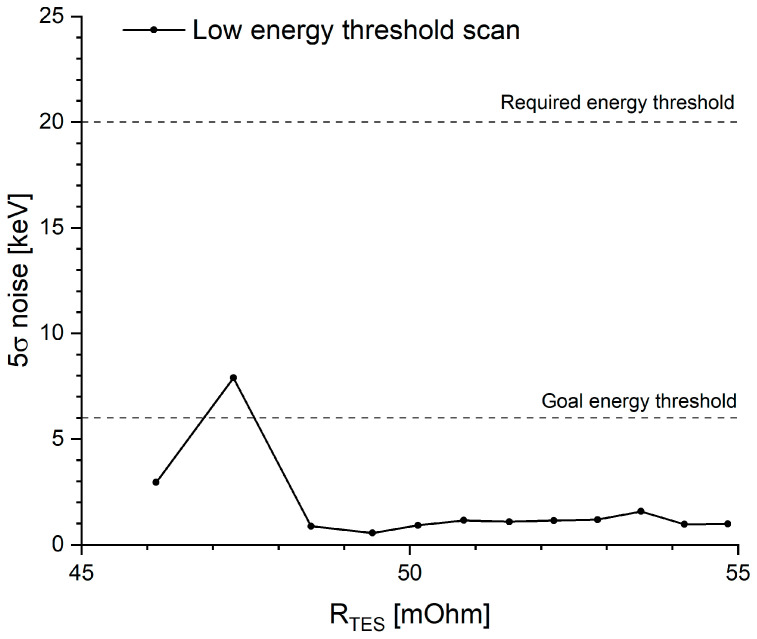
Energy threshold scan probed at TES mid-transition performed by using ^55^Fe source. 1-σ is the rms value of the baselines of the pulses, i.e., the noise in the pulse baseline.

The precise detector working point was selected by executing an energy threshold scan under ^55^Fe illumination (see Figure 14). At the optimized working point (I_bias_~750 μA, R_TES_~49 mΩ), the device achieved a low-energy threshold of ~0.6 keV (amply compliant with the requirement of <20 keV, and the goal of <6 keV) while dissipating only 5.15 nW of power at 50 mK (requirement < 10 nW), including the SQUID dissipation evaluated at its working point as 1.0 nW.

#### 3.3.2. Performance Under Source Illumination and Particle Background

For verifying TRL5, it was decided to back-illuminate the detector with respect to the TES position with a ^55^Fe source, and subsequently acquire a background spectrum in the laboratory. From these, we extracted useful information regarding the low-energy threshold, spectral resolution, and timing. The back-illumination of the chip ensures that the detector responds efficiently even to events generated at the point farthest from the sensor (the TES location is far from the photon absorption centre that in silicon is within ~80 µm, where the phonon bubble is generated and subsequently decays in the thermal regime). The combination of results from the radioactive source and the environmental background demonstrates a solid control of the technologies critical to chip development within the context of TRL5 verification.

**Figure 15:**
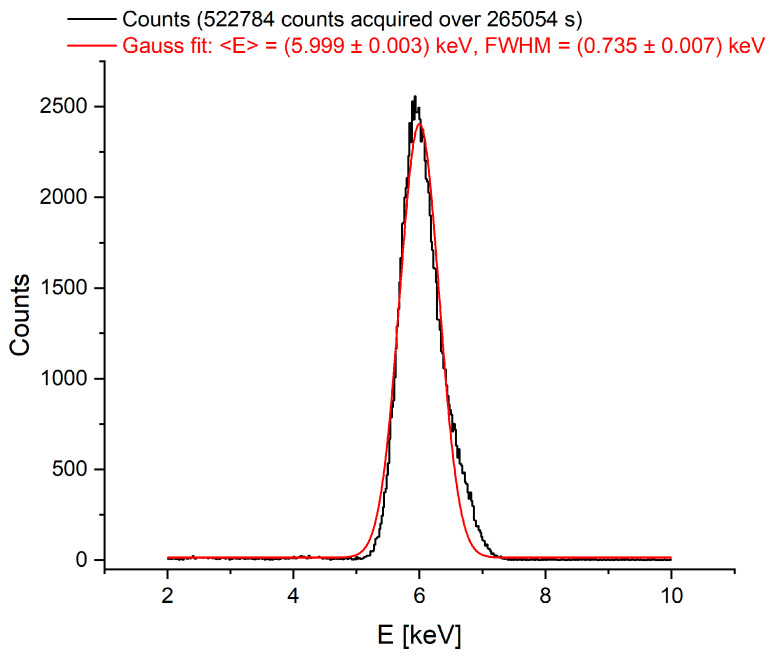
DM1.2#3 Energy spectrum (raw Pulse Height used as energy estimator and calibrated with the peak at 6 keV of the ^55^Fe source). The slight excess around 6.5 keV is due to the unresolved K_β_ line, which broadens the main K_α_ line at ~6 keV. The laboratory background is negligible and contributing to ~0.4% (evaluated from the 6 keV energy bin shown in Figure 16).

For spectral analysis, by using a ^55^Fe source (~6 keV), we got an energy resolution of ΔE_FWHM_ = 735 eV @ 6 keV (Figure 15). It is worth noting that, as anticoincidence detector, it is not required to produce a spectroscopic detector, though it is interesting having a CryoAC performing in such a way [16].

**Figure 16:**
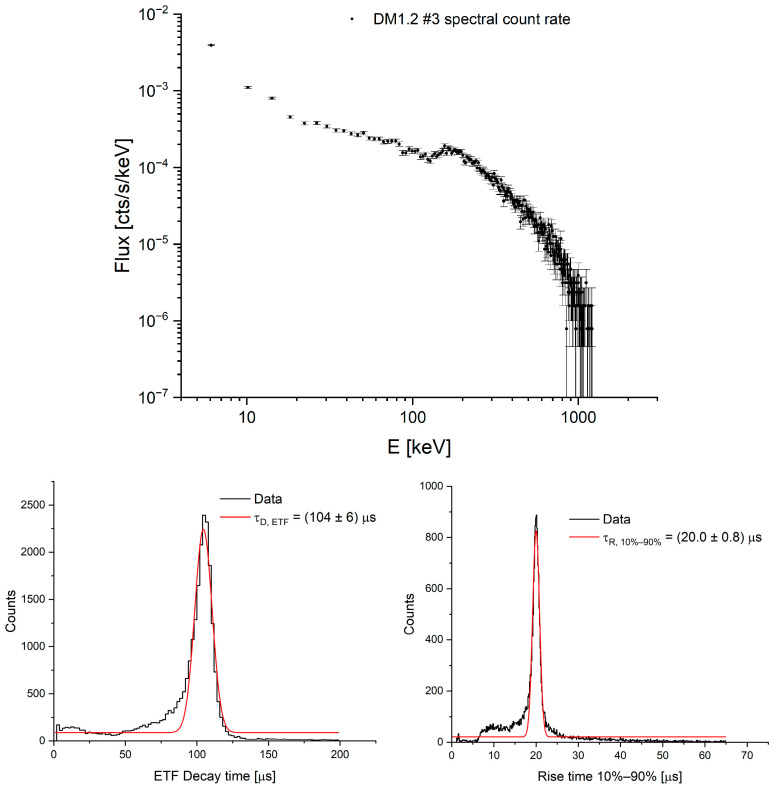
(**Top**): Spectral count rate (raw Pulse Height) of background events detected by the CryoAC DM1.2#3: the value of the acquired signal in Volt related to the peak of the MIP, has been matched to the 150 keV value expected from Geant4 simulation. (**Bottom**) Thermal decay and 10–90% rise time distribution of the background events.

Background events were acquired with the assembly placed parallel to the floor to maximize the capture rate of Minimum Ionizing Particles (MIP/muons). The spectrum shown in Figure 16 has been produced by matching the peak of the MIP from the acquired signal in V to the expected ~150 keV value related to the most probable deposited energy in silicon 500 μm thick, as expected from Geant4 simulation. About the position of the peak energy, we could have a systematic of ~13% as recorded on the previous detector [17], for which we are improving the definition of the Geant4 mass model to better understand the origin of this discrepancy; however, such an uncertainty is fully acceptable for the CryoAC detector whose primary role is anticoincidence vetoing, not spectroscopy. If the decision were taken at system level, it would be interesting to monitor the temporal variations in the particle flux, which makes any systematic error in the absolute energy irrelevant. As shown, the broad Landau distribution is successfully resolved. At lower energy, the spectrum increases due to secondaries and environmental gammas. The timing characteristics extracted from these high-energy background events confirmed a highly responsive 10–90% rise time of 20 μs, and an ETF decay time of 100 μs. Raw data analysis results are shown in Figure 16.

## 4. Discussion

The unified analysis of microfabrication constraints and cryogenic performance confirms the high maturity of the DM1.2 design.

Single process verification (Ir deposition thickness homogeneity, TES RIE vs. T_C_, Si DRIE affecting wide area suspended absorbers) campaign results have shown a very stable production process, culminating in the production of 3 DM1.2 samples.

The critical temperature shift observed in sample #3 (see Figure 12) was traced back to Ir target conditioning during the thin film stage deposition. The fabrication team determined that after six consecutive Iridium runs, the deposition rate increases significantly due to surface modifications of the pill and, as a direct mitigation strategy for future batches, production flights will be restricted to a maximum of six consecutive depositions, thus restoring the strict reproducibility observed between samples #1 and #2.

When driven within its operational SQUID FLL configuration, the DM1.2 #3 pixel satisfied every CryoAC detector requirement/goal, as summarized in Table 4 though having a transition curve not compliant with the test pass criteria based on design values (see Figure 12 and Table 3).

The low-energy threshold of 0.6 keV is significantly better than the 6 keV goal, ensuring that the CryoAC can effectively flag low-energy background events. Further, fast time constants make it possible to keep the CryoAC deadtime under control.

Though we do not have verified performance on sample #1 and #2, we are satisfied of the results because sample #3, which features a non-nominal transition, reaches CryoAC requirements/goal.

The shape of the transition sample #3 does not appear to be critical to performance. It is merely for comparison with the other two ones. For CryoAC aims in the context of the X-IFU performance, by taking into account all the activity performed so far, this means that we could relax the requirement related to the critical temperature (T_C_), and transition width (ΔT), whose values are at present under investigation.

About the yield assessment, by taking into account the criteria shown in Table 3, the yield is 2/3 because sample #3 does not match both the transition width value and the designed Rn.

However, since sample #3 was the only one tested in terms of performance, and the related goals/reqs criteria are amply met, we are inclined to relax the transition requirements as reported above. Adopting this formulation, the yield would be 3/3.

## 5. Programmatics

The achievement of the TRL5 milestone is the closure of a path that foresees in the X-IFU context a TRA (Technology Readiness Assessment) process subdivided into 2 parts, where the teams, developing critical technology, have to report progress with respect to what was reported in their related technology demonstration plan to CNES (X-IFU prime), to ESA, and, in our case, also to SRON being responsible for the X-IFU FPA.

Our first intermediate progress meeting was held in October 2025. The achievement of TRL5 of CryoAC will be formally validated at the TRA final review planned in Q4-2026.

The next NewAthena major step is the Mission Adoption, whose review will span within October 2026 and February 2027, for a planned closure milestone (achievement) in June 2027.

As far as the planning of the CryoAC project is concerned by 2028, after the closure of the TRA process, the major milestone will be the formal delivery of the following cold stage assembly models to the SRON team:

- the STM3, a form-fitting Structural and thermal model, mainly based on the one reported in Figure 3, to be thermal-cycled in 4K–300K range and sine/random vibrated, aimed at thermal and mechanical validation of the CryoAC Cold Stage toward the EM design, and vibrations within the STM FPA
- the EM, the Engineering Model aimed at TRL6 achievement
- the EQM, the Engineering Qualification Model aimed at FPA-level environmental qualification (thermal cycles and random vibrations) and FPA-level functional and performance tests only to verify survival of environmental tests

Further on, to de-risk the design of the EM CryoAC (TRL6) is at present ongoing the internal activity related to the so-called FSDeMo CryoAC (Full Scale Demonstration Model), which is a chip having hexagonal geometry and two-out-of-four pixels instrumented aiming at evaluating thermal cross-talk among the pixels, chip power dissipation, etc. The chip manufacturing has taken advantage of the validated technologies from DM1.2 in the context of the TRL5 phase here discussed. This activity is planned to be closed in Q4/2026.

For the sake of completeness, we would like to point out that from the structural point of view in the past we carried out two test campaigns in mechanical environment by vibrating two etched, 4-pixel hexagonal chips in different configuration under qualification vibrational masks provided by SRON, as follows:

- SM0, which was a chip glued on 3 pillars to assess whether the basic DRIE process affects in some way the structural property of the chip (successfully passed [18])
- SM1 (assembly made of chip + PCB + bracket) to understand the response of the assembly in mechanical environment (not published, internal report, chip not broken).

## 6. Conclusions

The full path from microfabrication process standardization to cryogenic functional verification toward TRL5 has been completed and verified.

Revising the standard operating procedures and cleanroom controls minimized old structural drifts (Section 2.1, Section 3.1 and Section 3.2). The cryogenic test campaign demonstrated that the DM1.2#3 complies with the required low-energy thresholds, power dissipation, and pulse timing speed parameters (Section 3.3). As far as the path to the TRL5 is concerned, all the demonstration activities have been successfully completed. With a clear fabrication mitigation plan established to ensure baseline repeatability, this milestone successfully completes the evaluation required to advance the CryoAC cryogenic subsystem toward official TRL5 status, to be formally validated at TRA final review planned in Q4-2026.

## Figures and Tables

**Table 1:** Uniformity of the deposited area and average thickness values over the 5 samples produced. Film #1, #2, #3, #5 comply with the required successful criteria. Film #4 was rejected as having a too- thick maximum value with respect to the average one.

Sample	Uniformity	Thickness
Film #1	1.61 ± 0.04 cm^2^	150 ± 1 nm
Film #2	1.53 ± 0.03 cm^2^	136 ± 1 nm
Film #3	1.63 ± 0.03 cm^2^	134 ± 1 nm
Film #4	1.71 ± 0.03 cm^2^	160 ± 1 nm
Film #5	1.69 ± 0.04 cm^2^	151 ± 1 nm

**Table 2:** Success criteria and measured value for the highlighted parameters.

	Average Vertical Angle	Scallop Depth	Scallop Height	Grass	Notch	Footing
Success	90° ± 1°	≤1 μm	2 μm	No	≤5 μm	≤5 μm
Sample #1	89°	~1 μm	~2 μm	No	<4 μm	<5 μm
Sample #2	89°	~1 μm	~2 μm	No	<5 μm	<4 μm
Sample #3	89°	~1 μm	~2 μm	No	<4 μm	5 μm

**Table 3:** Transition and heater parameters for the three DM 1.2 samples. Uncertainty on T_C_ is from calibration error, while that on ΔT is statistical.

Deposition Order	Sample Name	Tc (mK) [Design: 100 mK]	ΔT (mK) [Req.: <10 mK]	Rn (mΩ) [Design: 200 mΩ]	Rheater (Ω) [Goal: 50 Ω]
1°	DM 1.2 #1	101.0 ± 2.0	0.60 ± 0.14	180.30 ± 0.20	34.95 ± 0.05
2°	DM 1.2 #2	100.6 ± 2.0	0.70 ± 0.14	189.89 ± 16.6	45.80 ± 0.15
3°	DM 1.2 #3	115.8 ± 2.0	14.3 ± 0.14	100.43 ± 0.52	80.30 ± 0.16

**Table 4:** DM1.2 #3 sample measured parameters and CryoAC reqs/goals. About Rise time and ETF decay time, average distribution values are reported.

Parameter	CryoAC DM1.2 #3	CryoAC Goals/Reqs
Low-Energy Threshold	~0.6 keV	<6 keV (goal), <20 keV (req)
Bias current	0.758 mA	<1 mA (goal), <3 mA (req)
Power dissipation @ 50 mK	~5.15 nW	<10 nW/pixel (req)
Rise Time (10–90%)	20.0 ± 0.8 μs	<33 µs (goal)
ETF decay time	104 ± 6 μs	<250 µs (goal)
ΔE_FWHM_ @ 6 keV	735 ± 7 eV	<2500 eV (goal)

## Data Availability

The data that support the findings of this study are available from the corresponding author upon reasonable request.
